# Arrhythmogenic mechanisms of interleukin-6 combination with hydroxychloroquine and azithromycin in inflammatory diseases

**DOI:** 10.1038/s41598-022-04852-5

**Published:** 2022-01-20

**Authors:** Xiaojia Zhu, Yuwei Wang, Yujie Xiao, Qianwen Gao, Li Gao, Wenhui Zhang, Xiaofeng Xin, Kesu Chen, Ujala Srivastava, Vamsi Krishna Murthy Ginjupalli, Michael Cupelli, Pietro Enea Lazzerini, Pier Leopoldo Capecchi, Long Chen, Mohamed Boutjdir

**Affiliations:** 1 National Standard Laboratory of Pharmacology for Chinese Materia Medica, School of Pharmacy, Nanjing University of Chinese Medicine, Nanjing, 210036 China; 2 Department of Respiration, Affiliated Jinling Hospital School of Medicine, Nanjing University, Nanjing, 210002 China; 3 Research and Development (151), Cardiovascular Research Program, Departments of Medicine, Cell Biology and Pharmacology, VA New York Harbor Healthcare System, State University of New York Downstate Health Sciences University, Brooklyn, NY 11209 USA; 4 Department of Medical Sciences, Surgery and Neurosciences, University of Siena, Siena, Italy; 5 Institute of Chinese Medicine of Taizhou China Medical City, Taizhou, 225300 China; 6 Department of Medicine, New York University School of Medicine, New York, NY USA

**Keywords:** Drug discovery, Immunology, Physiology, Cardiology, Medical research

## Abstract

Inflammatory diseases including COVID-19 are associated with a cytokine storm characterized by high interleukin-6 (IL-6) titers. In particular, while recent studies examined COVID-19 associated arrhythmic risks from cardiac injury and/or from pharmacotherapy such as the combination of azithromycin (AZM) and hydroxychloroquine (HCQ), the role of IL-6 per se in increasing the arrhythmic risk remains poorly understood. The objective is to elucidate the electrophysiological basis of inflammation-associated arrhythmic risk in the presence of AZM and HCQ. IL-6, AZM and HCQ were concomitantly administered to guinea pigs in-vivo and in-vitro*.* Electrocardiograms, action potentials and ion-currents were analyzed. IL-6 alone or the combination AZM + HCQ induced mild to moderate reduction in heart rate, PR-interval and corrected QT (QTc) in-vivo and in-vitro. Notably, IL-6 alone was more potent than the combination of the two drugs in reducing heart rate, increasing PR-interval and QTc. In addition, the in-vivo or in-vitro combination of IL-6 + AZM + HCQ caused *s*evere bradycardia, conduction abnormalities, QTc prolongation and asystole. These electrocardiographic abnormalities were attenuated in-vivo by tocilizumab (TCZ), a monoclonal antibody against IL-6 receptor, and are due in part to the prolongation of action potential duration and selective inhibition of Na^+^, Ca^2+^ and K^+^ currents. Inflammation confers greater risk for arrhythmia than the drug combination therapy. As such, in the setting of elevated IL-6 during inflammation caution must be taken when co-administering drugs known to predispose to fatal arrhythmias and TCZ could be an important player as a novel anti-arrhythmic agent. Thus, identifying inflammation as a critical culprit is essential for proper management.

## Introduction

Severe acute respiratory syndrome coronavirus-2 (SARS-CoV-2) causes the human respiratory illness COVID-19, and is a serious global pandemic. COVID-19 has inflected major death tolls worldwide^1,2^. While COVID-19 presents as an acute respiratory infection, it also affects other organs, including the heart. Only recently studies examining COVID-19 associated cardiac arrhythmic risks from direct cardiac injury and/or from pharmacotherapy became available^3–8^. However, following COVID-19 infection, the role of the cytokine storm, namely the interleukin-6 (IL-6) in increasing the arrhythmic risk remains poorly understood^9^. This arrhythmic risk is a major concern as it is compounded by the use of drugs known to predispose to cardiac arrhythmias. Several drugs were used in the treatment of COVID-19 and are still currently used in other autoimmune/inflammatory diseases. The most controversial is the antimalarial hydroxychloroquine (HCQ)^10–13^ intriguingly still in use in several countries for COVID-19^14^ and also for the treatment of rheumatoid arthritis and systemic lupus erythematosus and its combination with the commonly used antibiotic azithromycin (AZM)^8,15–17^ both of which are listed at crediblemeds.org as definite causes of the long QT-associated polymorphic ventricular arrhythmia known as Torsade de Pointes (TdP).

Elevated IL-6 levels were reported in severely affected COVID-19 patients^18–20^ and correlated with high mortality^18^. As such, IL-6 receptor (IL-6R) inhibitors such as tocilizumab (TCZ), a monoclonal antibody against the IL-6R, are being explored for the treatment of the cytokine storm^21–25^, indicating a potential of a previously unrecognized direct electrophysiological role of IL-6 in the arrhythmogenesis of COVID-19 unrelated to its proinflammatory properties^9,26^. In fact, accumulating recent evidence demonstrated that inflammatory cytokines, particularly IL-6, significantly increase the risk of QTc prolongation and TdP^27^ via direct modulating effect on cardiac ion channel’s function^28–30^. Based on these evidences, understanding the consequences of the concomitant presence of risks factors (IL-6 + AZM + HCQ) on the arrhythmic risk, is urgently needed to inform the clinical decisions for not only COVID-19 patients but also for patients with other autoimmune/inflammatory conditions. Here, we establish the electrophysiological basis underlying the arrhythmias seen in patients with COVID-19, using in-vivo and in-vitro guinea pigs treated with the combination of IL-6, AZM and HCQ. The data also demonstrate the potential and novel role of TCZ as an anti-arrhythmic agent for inflammatory conditions in which other drugs known to predispose to arrhythmia are concomitantly used.

## Results

### In-vivo impact of the combination of IL-6, hydroxychloroquine (HCQ), and azithromycin (AZM) on guinea pig ECG

**Figure 1:**
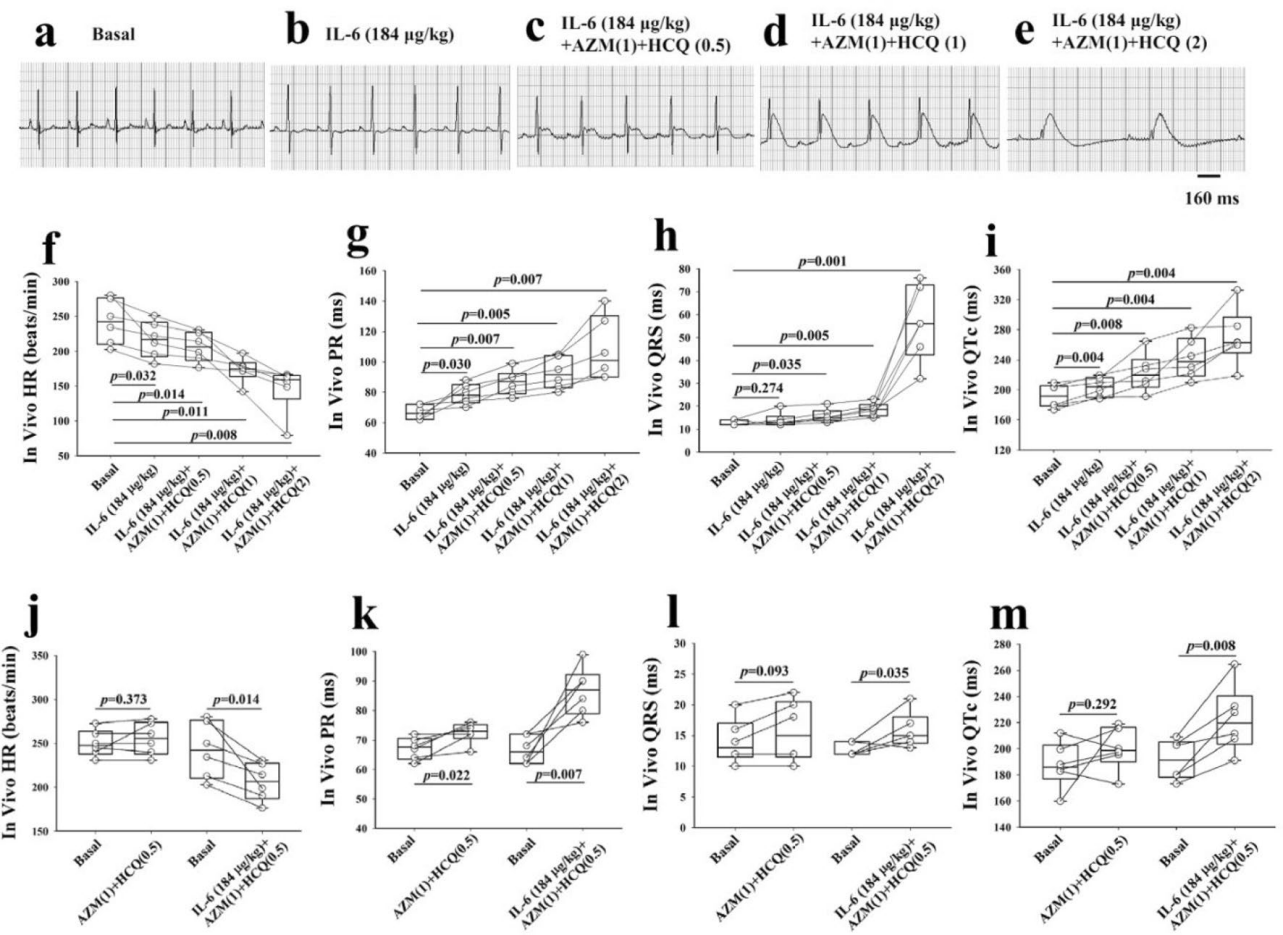
In-vivo impact of interleukin-6, azithromycin and hydroxychloroquine on the electrocardiogram of guinea pigs. (**a**) ECG of a guinea pig before, (**b**) after administration of IL-6(184 μg/kg) alone, (**c**) IL-6 + AZM(1) + HCQ(0.5), (**d**) IL-6 + AZM(1) + HC(1) and (**e**) IL-6 + AZM (1) + HCQ(2). Comparison of individual data points for heart rate (HR), PR, QRS and QTc between baseline and different interventions is illustrated in panels (**f–i)** using paired *t*-test versus basal group and one-way repeated measures analysis of variance for multiple comparisons among the drug interventions. Heart rate (**j**), PR (**k**), QRS (**l**) and QTc (**m**) data from two groups of guinea pigs, one (n = 6) injected with AZM (1) and HCQ (0.5) and the other (n = 6) injected with IL-6 (184 μg/kg) + AZM (1) + HCQ (0.5). AZM (1) = 38.2 mg/kg; HCQ(0.5) = 22.9 mg/kg; HCQ(1) = 45.8 mg/kg; HCQ(2) = 91.6 mg/kg. *p* values shown are in comparison to basal conditions.

**Figure 2:**
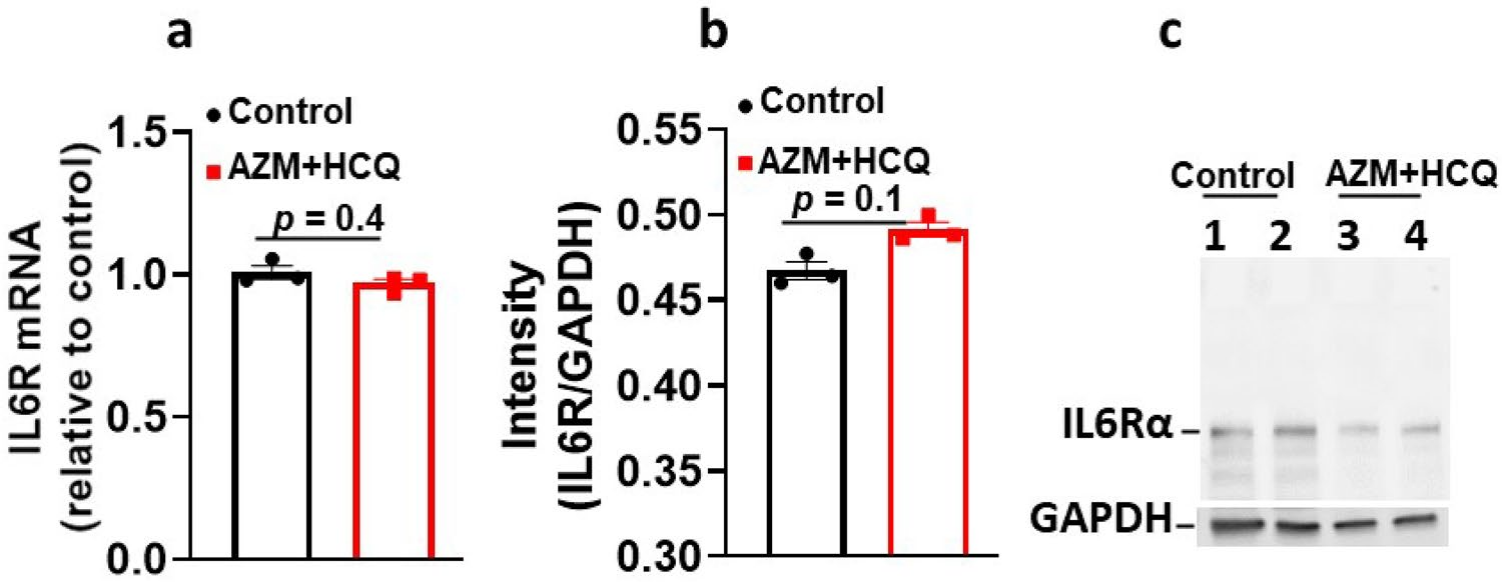
In vivo impact of azithromycin and hydroxychloroquine on the IL-6R transcript and proteins levels. (**a**) mRNA levels of IL6R in untreated guinea pig hearts (control) and in guinea pigs treated with AZM (1) and HCQ (2) for 30 min. (**b**) Densitometric analysis and (**c**) the corresponding western blot for IL6Rα proteins untreated guinea pig heart lysate (control) and guinea pigs treated with AZM (1) and HCQ (2) for 30 min. The blots were probed with a monoclonal antibody for IL6Rα (Anti IL6Rα—H7 Santa Cruz). The band at 80 kDa represents IL6Rα. GAPDH indicates internal control. Each experiment was performed in triplicates from 4 guinea pigs (2 controls, lanes 1 & 2 and 2 treated with AZM + HCQ, lanes 3 & 4).

Because IL-6, AZM and HCQ are known to individually affect multiple ion channels^28,31,32^ and because the net electrophysiological effect on the ECG is unknown, we set to first test these compounds in combination in-vivo and then evaluate their effects on the ECG. At baseline conditions, ECGs were first recorded from all the six, initially untreated, guinea pigs which served as their own control (Fig. 1a). Subsequently, guinea pigs were sequentially and cumulatively injected intravenously first with IL-6 (184 μg/kg) for 40 min to mimic the clinical settings where inflammation precedes drug therapy, followed by intravenous injection with AZM (1-time clinically relevant dose (CRD), 38.2 mg/kg) and intraperitoneal injection with HCQ (0.5-times CRD, 22.9 mg/kg) for 30 min followed by the cumulative injection of two higher dose of HCQ (1-time CRD, 45.8 mg/kg and 2-times CRD, 91.6 mg/kg) at 30 min intervals. ECGs were taken after each injection. IL-6 alone significantly reduced heart rate (Fig. 1b,f**)**, prolonged PR interval (Fig. 1b,g) and QTc (Fig. 1b,i). IL-6 combined with AZM and HCQ (0.5-time CRD) or HCQ (1-time CRD) or HCQ (2-time CRD) caused further significant and dose-dependent bradycardia, PR, QRS and QTc prolongations, and complete atrioventricular dissociation (Fig. 1c–i and Table 1A). Interestingly, the administration of only the combination of AZM and HCQ (0.5-time CRD) without IL-6 in another set of six guinea pigs, resulted in no effect on heart rate and QRS, lesser PR prolongation (ΔPR = 6 ms vs. 20 ms with IL-6) and non-significant QTc prolongation (ΔQTc = 12 ms vs. 31 ms with IL-6) indicating that IL-6 significantly amplifies the abnormal ECG phenotype (Fig. 1j–m and Table 1A, B). To assess whether AZM and HCQ combination may have per se affected IL-6R expression, we measured the corresponding mRNA and proteins levels. IL-6R mRNA levels in hearts of guinea pigs injected with AZM (1) (38.2 mg/kg) and HCQ (2) (91.6 mg/kg) showed no significant change in transcript levels of IL-6R (1.0-fold and 0.96-fold change when mRNA was normalized to control hearts, experiments were performed in triplicates, *p* = 0.4, Fig. 2a). Likewise, protein levels of IL-6R were measured in western blots and densitometric analysis showed no significant change between the control and guinea pigs treated with AZM and HCQ (the mean intensity for the control guinea pigs was 0.46 vs. 0.49 for the treated guinea pigs, experiments were performed in triplicates, *p* = 0.1, Fig. 2b,c and see supplementary Fig. S1 online).

**Table 1:** In-vivo impact of the combination of IL-6, HCQ, AZM, and TCZ on guinea pig ECG.

	HR (bpm)	PR (ms)	QRS (ms)	QTc (ms)
A. Basal	243 ± 13.0	67 ± 1.9	13 ± 0.4	192 ± 6.3
IL-6 (184 μg/kg)	218 ± 11.7*	79 ± 2.7*	14 ± 1.2	204 ± 5.3*
IL-6 (184 μg/kg) + AZM (1) + HCQ (0.5)	206 ± 8.7*	87 ± 3.4*	16 ± 1.2*	223 ± 10.4*
IL-6 (184 μg/kg) + AZM (1) + HCQ (1)	173 ± 7.4*,**,***	93 ± 4.3*,**	19 ± 1.2*	242 ± 11.2*,**
IL-6 (184 μg/kg) + AZM (1) + HCQ (2)	146 ± 13.7*,**,***,****	108 ± 8.5*,**,***,****	56 ± 6.7*,**,***,****	270 ± 15.3*,**,***,****
B. Basal	250 ± 6.1	67 ± 1.5	14 ± 1.5	188 ± 7.2
AZM (1) + HCQ (0.5)	255 ± 7.5	73 ± 1.5*	16 ± 2.0	200 ± 6.7
C. Basal	283 ± 22.3	52 ± 2.4	17 ± 1.4	186 ± 8.4
Tocilizumab (10 mg/kg)	284 ± 24.0	53 ± 1.4	17 ± 1.8	181 ± 11.3
Tocilizumab (10 mg/kg) + IL-6 (184 μg/kg)	290 ± 23.0	54.7 ± 1.8	18 ± 1.5	181 ± 11.6
Tocilizumab (10 mg/kg) + IL-6 (184 μg/kg) + AZM (1) + HCQ (0.5)	288 ± 17.4	54 ± 1.5	19 ± 1.4	195 ± 11.8**,***
Tocilizumab (10 mg/kg) + IL-6 (184 μg/kg) + AZM (1) + HCQ (1)	281 ± 18.2	56 ± 1.7	19 ± 1.5	208 ± 10.3**,***
Tocilizumab (10 mg/kg) + IL-6 (184 μg/kg) + AZM (1) + HCQ (2)	257 ± 14.4**,***,****,*****	59 ± 1.8*,**,***,****,*****	22 ± 1.9*,**,***,****,*****	224 ± 8.3*,**,***,****,*****

All values are expressed as mean ± SE, n = 6 guinea pigs for (A), n = 6 guinea pigs for (B) and n = 6 guinea pigs for (C). In panel (A), paired t-test was used to compare data before (basal) and after the drugs; each guinea pig is used as its own control. One-way repeated measures analysis of variance was used to compare data between the treatments with IL-6 alone and the combination with AZM and HCQ for HR, PR, QRS and QTc in the same group of guinea pigs. *p < 0.05 versus Basal; **p < 0.05 versus IL-6 (184 μg/kg); ***p < 0.05 versus IL-6 (184 μg/kg) + AZM (1) + HCQ (0.5);

****p < 0.05 versus IL-6 (184 μg/kg) + AZM (1) + HCQ (1). In panel (B), the paired t-test was used to compare data from AZM (1) + HCQ (0.5) for HR, PR, QRS and QRS with basal conditions. *p < 0.05 versus Basal. In panel (**C**), paired t-test was used to compare data before (basal) and after the drugs; each guinea pig is used as its own control. One-way repeated measures analysis of variance was used to compare data between the treatments with Tocilizumab alone and in combination with IL-6, AZM and HCQ for HR, PR, QRS and QTc in the same group of guinea pigs. *p < 0.05 versus Basal; **p < 0.05 versus Tocilizumab (10 mg/kg); ***p < 0.05 versus Tocilizumab (10 mg/kg) + IL-6 (184 μg/kg); ****p < 0.05 versus Tocilizumab (10 mg/kg) + IL-6 (184 μg/kg) + AZM (1) + HCQ (0.5); *****p < 0.05 versus Tocilizumab (10 mg/kg) + IL-6 (184 μg/kg) + AZM (1) + HCQ (1). The numbers in parenthesis indicate the corresponding multiple(s) of clinically relevant dose (CRD). QTc = QT/RR^1/3^. *HR* heart rate, *IL-6* Interleukin-6, *AZM* azithromycin, *HCQ* Hydroxychloroquine and *TCZ* Tocilizumab. AZM (1) = 38.2 mg/kg; HCQ (0.5) = 22.9 mg/kg; HCQ (1) = 45.8 mg/kg; HCQ (2) = 91.6 mg/kg.

**Figure 3:**
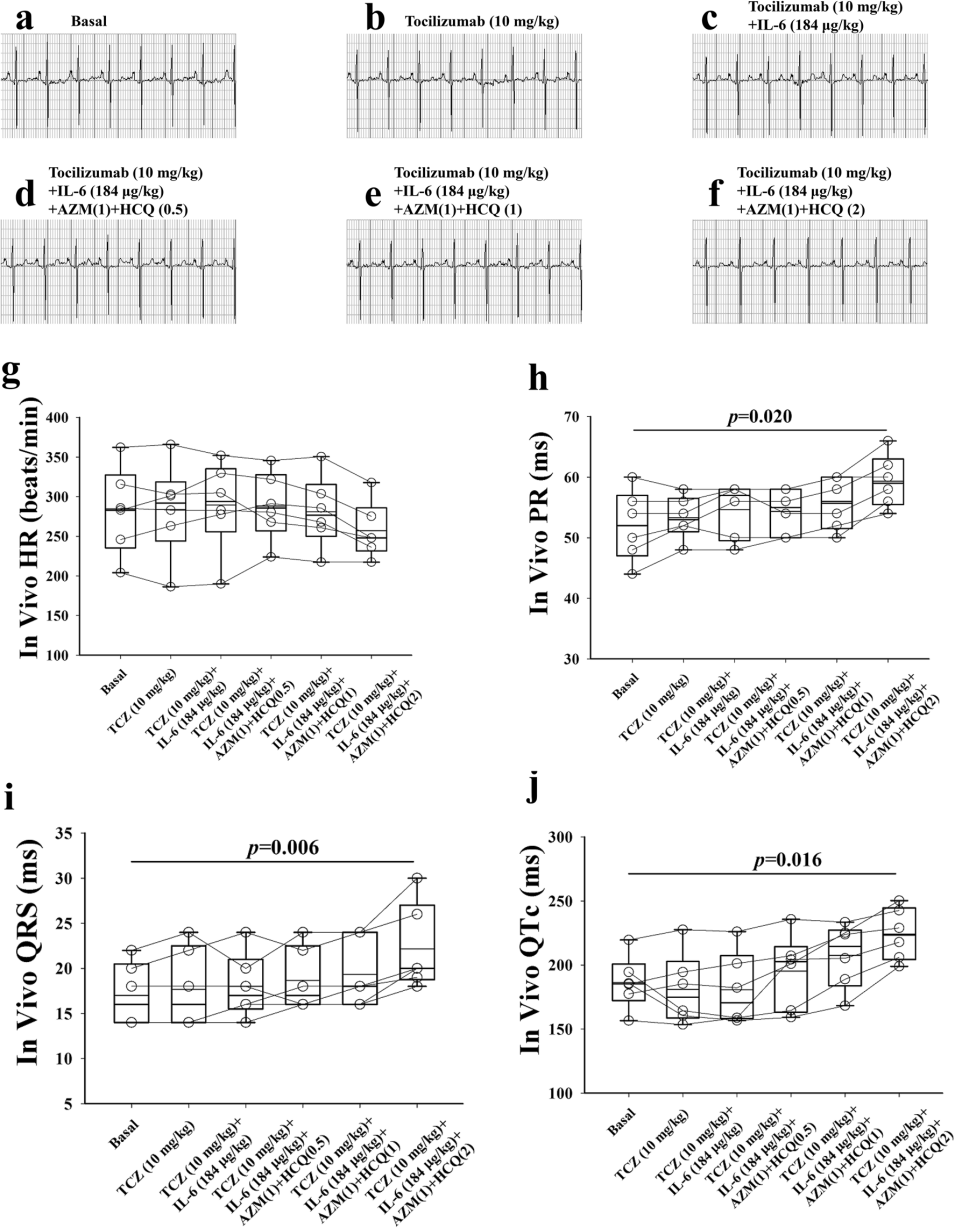
In-vivo impact of tocilizumab, interleukin-6, azithromycin and hydroxychloroquine on the electrocardiogram of guinea pigs (**a**) ECG of a guinea pig before, (**b**) after administration of TCZ (10 mg/kg) alone, (**c**) TCZ + IL-6(184 μg/kg), (**d**) TCZ + IL-6 + AZM(1) + HCQ(0.5), (**e**) TCZ + IL-6 + AZM(1) + HC(1) and (**f**) TCZ + IL-6 + AZM (1) + HCQ(2). Comparison of individual data points for heart rate (HR), PR, QRS and QTc between baseline and different interventions is illustrated in panels (**g–j**). AZM(1) = 38.2 mg/kg; HCQ (0.5) = 22.9 mg/kg; HCQ(1) = 45.8 mg/kg; HCQ(2) = 91.6 mg/kg. *p* values shown are in comparison to basal conditions.

To test whether IL-6 worsening of the electrocardiographic abnormalities of AZM and HCQ can be attenuated pharmacologically, TCZ (10 mg/kg), the IL-6R inhibitor, was administered intravenously in-vivo to six guinea pigs for 10 min first, followed by IL-6, AMZ and HCQ as outlined above. TCZ alone had no significant effects on heart rate (Fig. 3b,g), PR interval (Fig. 3b,h), QRS (Fig. 3b,i) and QTc (Fig. 3b,j) compared to basal conditions (Fig. 3a,g–j). However, TCZ prevented the IL-6 (184 μg/kg) from reducing heart rate, prolonging PR interval and QTc (Fig. 3c, g–j and Table 1C). Similarly, TCZ also prevented the combination of IL-6, AZM (1-time CRD) and HCQ (0.5 or 1-time CRD) from reducing the heart rate and prolonging PR interval and QRS, attenuated QTc prolongation but not significantly when using one-way repeated measures analysis of variance (Fig. 3 and Table 1C). However, TCZ prevented the atrioventricular dissociation induced by 2-times HCQ (Figs. 1e, 3f).

### In-vitro impact of the combination of IL-6, AZM and HCQ on Langendorff perfused guinea pig hearts

**Figure 4:**
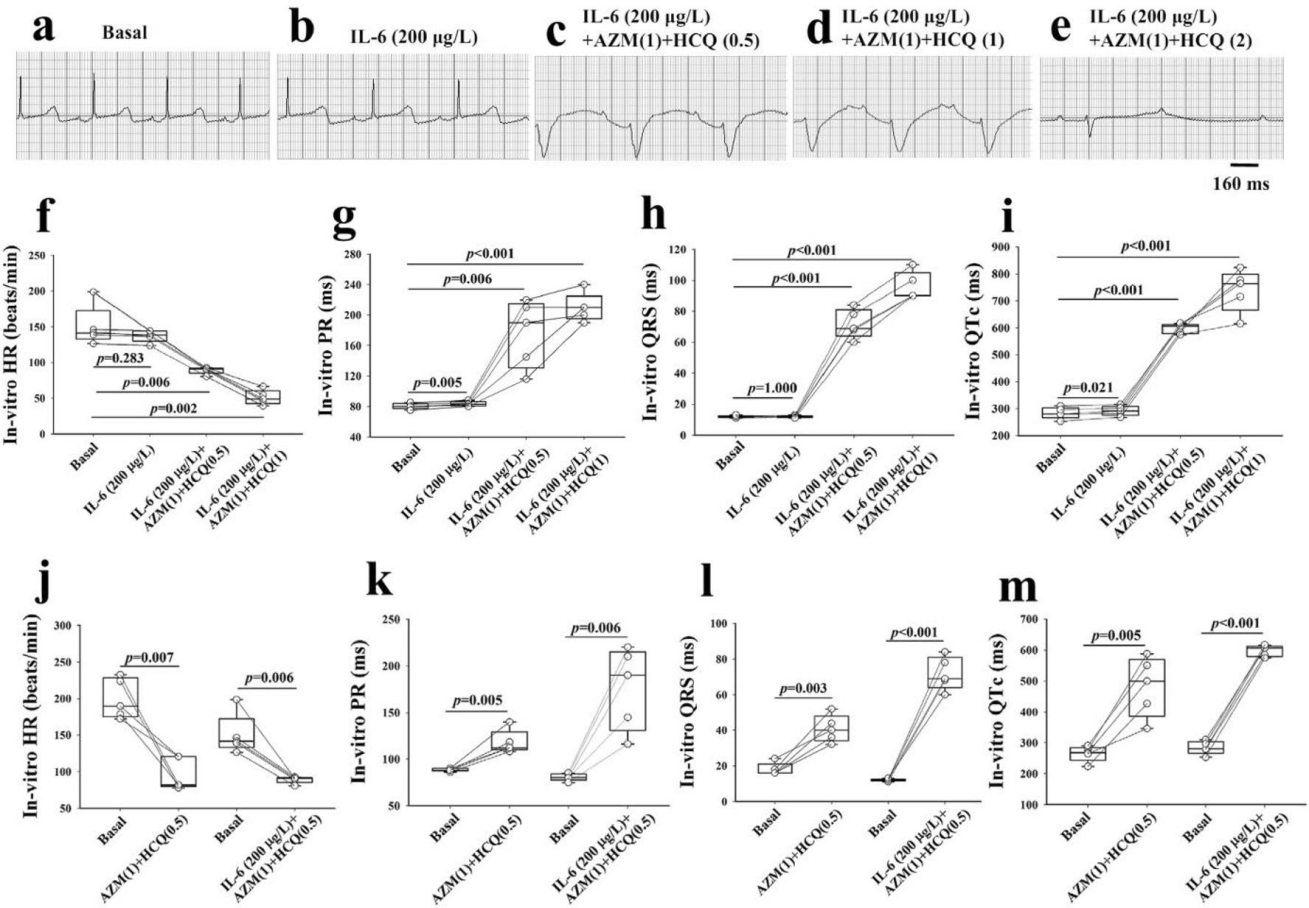
In-vitro impact of interleukin-6, azithromycin and hydroxychloroquine on the electrogram of Langendorff perfused guinea pig hearts. Electrograms of an isolated guinea pig heart before (**a**) and (**b**) after perfusions of IL-6 (200 μg/L) alone for 40 min, (**c**) IL-6 + AZM(1) + HCQ(0.5), (**d**) IL-6 + AZM(1) + HCQ(1) and (**e**) IL-6 + AZM (1) + HCQ (2). The comparison of individual data points for heart rate (HR), PR, QRS and QTc between baseline and the different interventions is illustrated in panels (**f–i)** using paired t-test. Heart rate (**j**), PR (**k**), QRS (**l**) and QTc (**m**) data from two groups of guinea pigs, one (n = 5) perfused with AZM(1) and HCQ(0.5) and the other (n = 5) perfused with IL-6(200 µg/L) + AZM(1) + HCQ(0.5). AZM(1) = 41.5 mg/L; HCQ(0.5) = 24.9 mg/L. AZM(1) = 41.5 mg/L; HCQ(0.5) = 24.9 mg/L; HCQ(1) = 49.8 mg/L; HCQ(2) = 99.6 mg/L. Paired t-test was used to compare interventions to baseline. (*) indicates *p* < 0.05.

Next, we aimed at assessing the *direct* impact of the combination of IL-6, AZM and HCQ on the heart by recording surface electrograms from isolated Langendorff perfused 5 guinea pig hearts (Fig. 4a). We first perfused the hearts with IL-6 (200 μg/L) alone for 40 min^28^, then cumulatively added AZM (1-time clinically relevant concentration (CRC), 41.5 mg/L) followed immediately by HCQ (0.5-time CRC, 24.9 mg/L) then by HCQ (1-time CRC, 49.8 mg/L) and HCQ (2-time CRC, 99.6 mg/L) at 8–10 min intervals. IL-6 resulted in significant PR (Fig. 4b,g) and QTc prolongations (Fig. 4b,i). The addition of AZM and HCQ (0.5-, 1- and 2-times CRC) to IL-6, resulted in a marked and significant concentration-dependent bradycardia, PR, QRS and QTc prolongations (Fig. 4c–i), followed by a complete atrioventricular dissociation and asystole in 5/5 hearts (Fig. 4e and Table 2A). Similar to the in-vivo studies above, the combination of only AZM and HCQ (0.5-time CRD) without IL-6 in another set of five guinea pigs, resulted in lesser PR prolongation (ΔPR = 30 ms vs. 95 ms with IL-6) and lesser QTc prolongation (ΔQTc = 218 ms vs. 314 ms with IL-6) indicating again that IL-6 amplifies the abnormal electrogram phenotype (Fig. 4j–m and Table 2A,B).

**Table 2:** In-vitro impact of the combination of IL-6, HCQ and AZM on Langendorff perfused guinea pig hearts.

	HR (bpm)	PR (ms)	QRS (ms)	QTc (ms)
A. Basal	150 ± 12.5	81 ± 1.7	12 ± 0.3	284 ± 9.6
IL-6 (200 μg/L)	138 ± 3.8	84 ± 1.4*	12 ± 0.3	292 ± 8.2*
IL-6 (200 μg/L) + AZM (1) + HCQ (0.5)	89 ± 2.1*,**	176 ± 19.8*,**	72 ± 4.2*,**	598 ± 8.1*,**
IL-6 (200 μg/L) + AZM (1) + HCQ (1)	51 ± 4.6*,**,***	210 ± 8.4*,**,***	96 ± 4.0*,**,***	739 ± 35.3*,**,***
IL-6 (200 μg/L) + AZM (1) + HCQ (2)	n/a	n/a	n/a	n/a
B. Basal	199 ± 12.1	88 ± 0.8	18 ± 1.5	264 ± 11.2
AZM (1) + HCQ (0.5)	96 ± 9.8*	118 ± 5.7*	41 ± 3.4*	482 ± 43.4*

All values are expressed as mean ± SE, n = 5 guinea pigs for (A) and n = 5 guinea pigs for (B). In panel (A), paired t-test was used to compare data before (basal) and after the drugs; each guinea pig heart is used as its own control. One-way repeated measures analysis of variance was used to compare data among the treatments of IL-6 alone and in combination with AZM and HCQ for HR, PR, QRS and QRS in the same group of isolated hearts. *p < 0.05 versus Basal; **p < 0.05 versus IL-6 (200 μg/L); ***p < 0.05 versus IL-6 (200 μg/L) + AZM(1) + HCQ (0.5). In panel (B), the paired t-test was used to compare data from AZM (1) + HCQ (0.5) for HR, PR, QRS and QRS with basal conditions. *p < 0.05 versus Basal. The numbers in parenthesis indicate the corresponding multiple(s) of clinically relevant concentration (CRC). QTc = QT/RR^1/3^. *HR* heart rate, *IL-6* Interleukin-6, *AZM* azithromycin, *HCQ* Hydroxychloroquine. AZM (1) = 41.5 mg/L; HCQ (0.5) = 24.9 mg/L; HCQ (1) = 49.8 mg/L; HCQ (2) = 99.6 mg/L. n/a: not applicable because of the asystole.

### In-vitro impact of the combination of IL-6, AZM and HCQ on action potentials of guinea pig left ventricular myocytes

**Figure 5:**
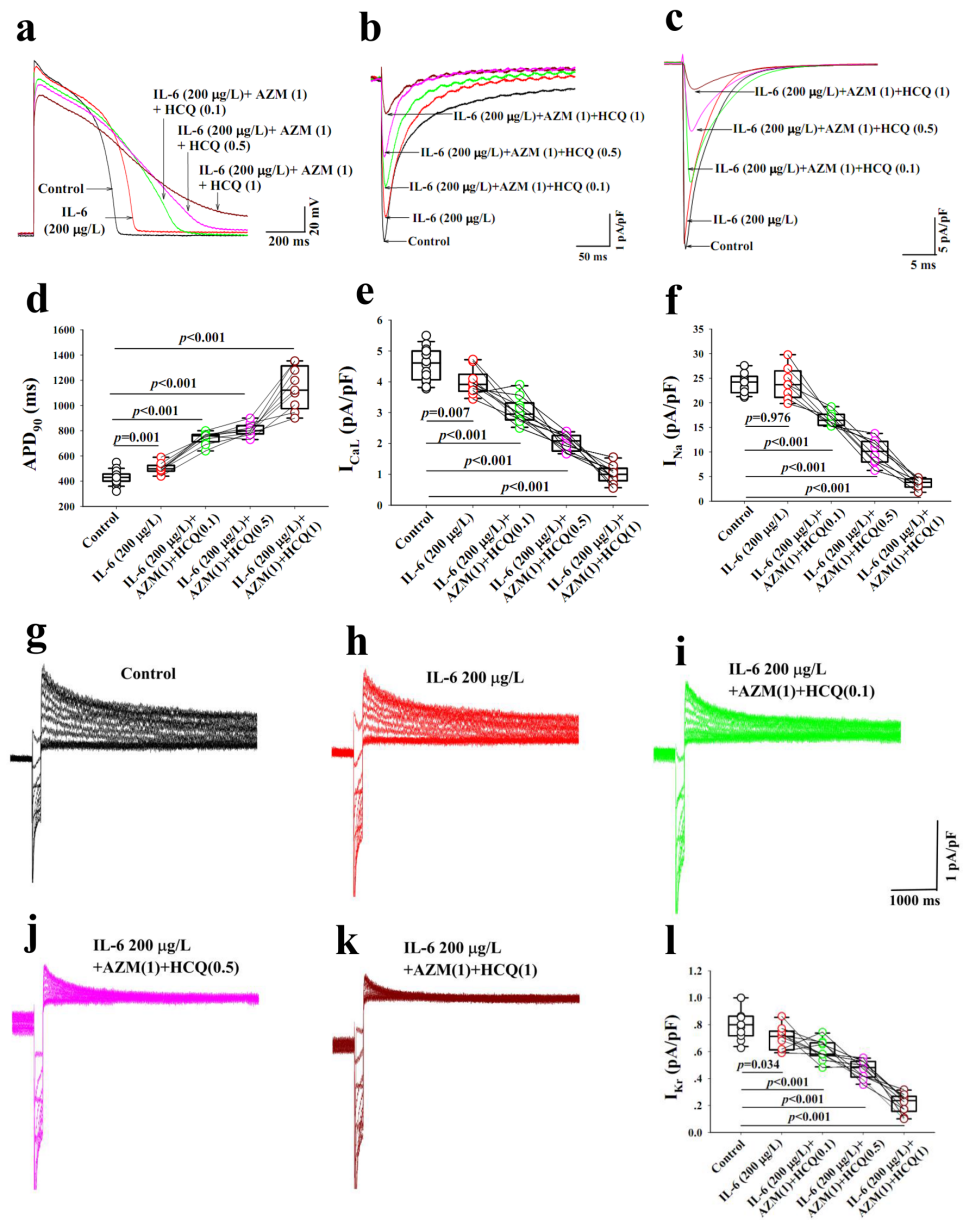
Impact of interleukin-6, azithromycin and hydroxychloroquine on action potential and key ion channels in guinea pig ventricular myocytes. Action potentials (**a**) and ion currents, I_CaL_ (**b**), I_Na_ (**c**) and I_Kr_ (**g–k**) recorded from control myocytes (black traces) and from another group of myocytes preincubated with IL-6 for 40 min (red traces), IL-6(200 µg/L) + AZM (1) + HCQ (0.1) (green traces), IL-6 + AZM (1) + HCQ(0.5) (pink traces) and IL-6 + AZM(1) + HCQ(1) (dark red traces). The comparison of individual data points for APD_90_ (**d**) and current densities for I_CaL_ (**e**), I_Na_ (**f**) and I_Kr_ (**l**) during control and after different interventions is presented. HCQ(0.1) = 5.0 mg/L; HCQ(0.5) = 24.9 mg/L; HCQ(1) = 49.8 mg/L; AZM(1) = 41.5 mg/L. Unpaired t-test was used between the control group and the group treated with IL-6 + AZM + HCQ. *p* values shown are comparison to basal conditions.

**Figure 6:**
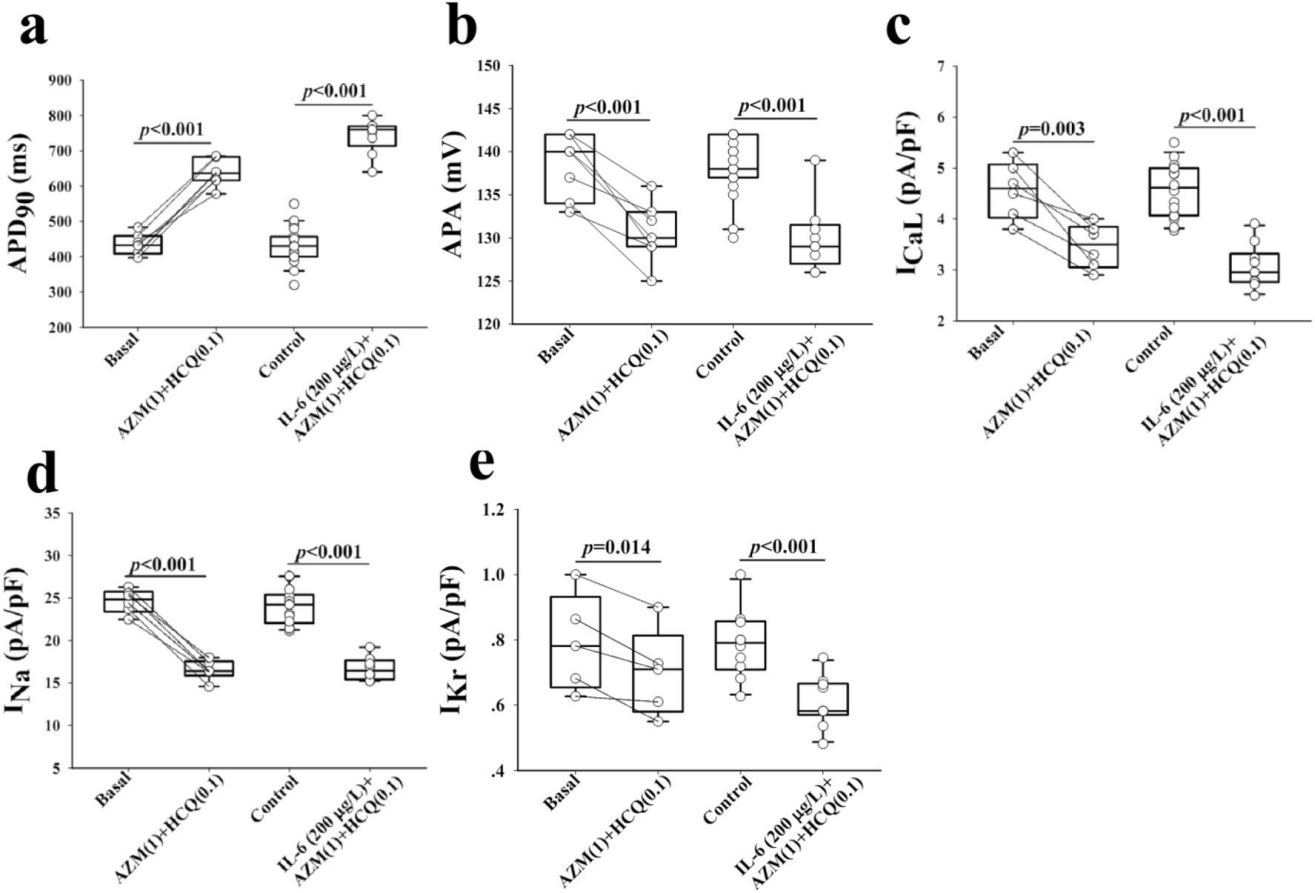
Impact of azithromycin and hydroxychloroquine with and without interleukin-6 on action potential and key ion channels in guinea pig ventricular myocytes. APD_90_ (**a**), AP amplitude (APA, **b**), I_CaL_ (**c**) I_Na_ (**d**) and I_Kr_ (**e**) data from two groups of guinea pig myocytes before (basal) and after administrations of AZM(1) + HCQ(0.1); and the other control group without IL-6 and the treated group with IL-6 (200 μg/L), AZM(1) and HQC(0.1). AZM(1) = 41.5 mg/L; HCQ(0.5) = 24.9 mg/L. Paired t-test was used within the same group before (basal) and with (AZM + HCQ). Unpaired t-test was used between control group and the group treated with IL-6 + AZM + HCQ. *p* values shown are comparison to basal conditions.

Next, we investigated the underlying mechanisms of the observed electrocardiographic abnormities seen in-vivo and in-vitro with the combination of IL-6, AZM and HCQ. The duration of AP is a surrogate for the QT interval and is impacted by I_Na_, I_CaL_ and I_Kr_ currents. As such the net impact of IL-6, AZM and HCQ on individual ion channels can be assessed accurately. The perfusion of IL-6 (200 μg/L) alone for 40 min resulted in a statistically significant prolongation of the APD_90_ without significant change in the AP amplitude (n = 9). The cumulative addition of AZM (1-times CRC, 41.5 mg/L), HCQ (0.1-time CRC, 5.0 mg/L) and HCQ (0.5-time CRC, 24.9 mg/L and 1-time CRC, 49.8 mg/L) resulted in a concentration-dependent prolongation of the APD_90_ and a decrease in AP amplitude (Fig. 5a,d)_._ The combination of IL-6 + AZM + HCQ when HCQ concentration reached 1-time CRC (49.8 mg/L) resulted in the failure of AP to completely repolarize (Fig. 5a and Table 3A). Note that HCQ at 2-times CRC (99.6 mg/L) in combination with IL-6 + AZM completely abolished AP and hence no related data are provided. Noteworthy again the perfusion of only the combination AZM and HCQ (0.1-time CRD) without IL-6, in another set of 7 myocytes, resulted in lesser APD_90_ prolongation (ΔAPD_90_ = 204 ms vs. 312 ms with IL-6) confirming that IL-6 amplifies the prolongation of APD_90_ and the reduction in AP amplitude (Fig. 6a,b and Table 3A,B).

**Table 3:** Impact of the combination of IL-6, AZM and HCQ on action potential, I_CaL_, I_Na_ and I_Kr_ of guinea pig left ventricular myocytes.

	APD_90_ (ms)	APA (mV)	I_CaL_ (pA/pF)	I_Na_ (pA/pF)	I_Kr_ (pA/pF)
A. Control	430 ± 11.5 (n = 19)	138 ± 0.8 (n = 19)	4.6 ± 0.17 (n = 16)	24.0 ± 0.56 (n = 14)	0.81 ± 0.04 (n = 11)
IL-6 (200 μg/L)	503 ± 14.1* (n = 9)	136 ± 1.3 (n = 9)	4.0 ± 0.13* (n = 10)	24.0 ± 1.16 (n = 8)	0.70 ± 0.03* (n = 10)
IL-6 (200 μg/L) + AZM(1) + HCQ (0.1)	742 ± 16.2*,** (n = 9)	130 ± 1.3*,** (n = 9)	3.1 ± 0.13*,** (n = 10)	16.7 ± 0.49*,** (n = 8)	0.61 ± 0.02*,** (n = 10)
IL-6 (200 μg/L) + AZM(1) + HCQ (0.5)	806 ± 16.7*,** (n = 9)	124 ± 1.6*,**,*** (n = 9)	2.0 ± 0.08*,**,*** (n = 10)	10.0 ± 0.90*,**,*** (n = 8)	0.47 ± 0.02*,**,*** (n = 10)
IL-6 (200 μg/L) + AZM(1) + HCQ (1)	1140 ± 56.3*,**,***,**** (n = 9)	119 ± 1.8*,**,***,**** (n = 9)	1.0 ± 0.09*,**,***,**** (n = 10)	3.6 ± 0.36*,**,***,**** (n = 8)	0.21 ± 0.02*,**,***,**** (n = 10)
B. Basal	433 ± 11.5 (n = 7)	138 ± 1.4 (n = 7)	4.6 ± 0.23 (n = 6)	24.6 ± 0.57 (n = 6)	0.79 ± 0.07 (n = 5)
AZM (1) + HCQ (0.1)	637 ± 14.3* (n = 7)	131 ± 1.3* (n = 7)	3.5 ± 0.18* (n = 6)	16.5 ± 0.47* (n = 6)	0.70 ± 0.06* (n = 5)

All values are expressed as mean ± SE. The number of experiments performed is indicated by “n = ”. Control conditions (A) refer to a separate group of cardiomyocytes versus basal conditions (B) which refer to the same group of cardiomyocytes used as their own control. In addition, one-way repeated measures analysis of variance was used to compare data between the treatments with IL-6 alone and the combination of AZM and HCQ for AP, APA, I_CaL_, I_Na_ and I_Kr_ in the same group of cardiomyocytes. *p < 0.05, versus Control; **p < 0.05, versus IL-6 (200 μg/L); ***p < 0.05, versus IL-6 (200 μg/L) + AZM(1) + HCQ (0.1); ****p < 0.05 versus IL-6 (200 μg/L) + AZM(1) + HCQ (0.5). In (B), the paired t-test was used to compare data from AZM (1) and HCQ (0.1) for AP, APA, I_CaL_, I_Na_ and I_Kr_ with basal conditions (*p < 0.05 vs. Basal). The numbers in parenthesis next to the drugs indicate the corresponding multiple(s) of clinically relevant concentration (CRC). *IL-6* Interleukin-6, *AZM* azithromycin, *HCQ* Hydroxychloroquine. AZM (1) = 41.5 mg/L; HCQ (0.1) = 5.0 mg/L; HCQ (0.5) = 24.9 mg/L; HCQ (1) = 49.8 mg/L.

### In-vitro effects of the combination of IL-6, AZM and HCQ on I_CaL_***, I***_Na_ and I_Kr_ on guinea pig left ventricular myocytes

Because the observed ECG and AP effects of the combination of IL-6, AZM and HCQ caused conduction delays, QTc and APD_90_ prolongations, we focused on characterizing the corresponding currents such as I_Na_, I_CaL_ and I_Kr_, which can affect conduction, APD and also QT interval respectively. Pre-incubation of IL-6 (200 μg/L) alone for 40 min significantly reduced densities of I_CaL_ (Figs. 4e, 5b and Table 3A) and I_Kr_ (Fig. 5h,l and Table 3A) without significant change in I_Na_ density (Fig. 5c/5f. and Table 3A). Myocytes pre-incubated with IL-6 and subsequently superfused with AZM (1-times CRC, 41.5 mg/L) and HCQ (0.1 or 0.5 or 1-times CRC, 5.0 or 24.9 or 49.8 mg/L) for 5–8 min periods for each concentration, significantly decreased I_CaL_, I_Na_ and I_Kr_ densities in a concentration-dependent manner (Fig. 5b,c,e–l and Table 3A). Finally, the perfusion of only the combination AZM and HCQ (0.1-time CRD) without IL-6, in another two sets of 6 and 5 cardiac myocytes, resulted in lesser reduction in I_CaL_ density (ΔI_CaL_ = 1.1 pA/pF vs. 1.5 pA/pF with IL-6) and similarly lesser reduction in I_Kr_ densities (ΔI_Kr_ = 0.09 pA/pF vs. 0.2 pA/pF with IL-6). Because IL-6 did not have any significant effect on I_Na_, no conclusion for this current was drawn (Fig. 6d). Collectively, IL-6 continued to amplifies the inhibitory effect on both I_CaL_ and I_Kr_ (Fig. 6c,e and Table 3A,B).

## Materials and methods

### Animal and ethical approval

Equal number of female and male Dunkin-Hartley guinea pigs (300–350 g) aged 4–5 weeks from the experimental animal center of Nanjing University of Chinese Medicine (Nanjing, China) were used in this study. All experiments involving animals were approved by the Animal-Care and Use-Committee of Nanjing University of Chinese Medicine (#202005A045), whose policies adhere to the USA National Institutes of Health Guide for the Care and Use of Laboratory Animals. The study was carried out in compliance with the ARRIVE guidelines.

### Chemicals

Hydroxychloroquine Sulfate (HCQ) was purchased from GLPBIO (USA, batch number: GC125441). Recombinant human interleukin-6 (IL-6) was purchased from Pepro TECH (USA, catalog number 200-06). Tocilizumab (TCZ) with a purity of more than 98.85% was purchased from MedChem Express (MCE, USA, batch number: 79456). Azithromycin lactobionate (AZM) was purchased from Yangtze River Pharmaceutical (Group) Co., Ltd (China, batch number: 19111691). All reagents were used on the same day of the experiment.

### Conversions of doses and concentrations between human and guinea pig

AZM, following prescribing specification, is used in clinical settings at a maximum of 500 mg/day intravenously for a human adult, which is equivalent to 8.3 mg/kg/day based on a body weight of 60 kg for a human adult. For in-vivo experiments, in accordance with a 4.6 times dose conversion factor between human and guinea pig based on body surface area^32–34^, a clinically relevant dose (CRD) of AZM was matched to 38.2 mg/kg for guinea pigs. Considering that human extracellular fluid is 20% of body weight, a human dose of azithromycin at 8.3 mg/kg is converted to a one-time clinical concentration of 41.5 mg/L for human subjects (based on intravenous injection, rapidly administered in the absence of any metabolites). For in-vitro experiments, 41.5 mg/L of AZM was utilized as one time clinically relevant concentration (CRC) for isolated heart and single cell experiments.

HCQ, following prescribing specification, is used in clinical settings at a maximum of 600 mg/day orally for a human adult. Based on the calculations above, the CRD for HCQ was calculated to be 45.8 mg/kg and the CRC was 49.8 mg/L.

IL-6 at 200 ng/ml (200 µg/L) was used in this study because lower concentrations at 20 ng/ml had no major effects on in-vivo and in-vitro studies (data not shown). Similarly, based on the fact that the 41.5 mg/L of CRC is equal to 38.2 mg/kg of CRD for AZM, 200 µg/L of CRC in-vitro was matched to 184 µg/kg of CRD for IL-6 in-vivo experiment in guinea pigs.

TCZ at 10 mg/kg was used for in-vivo ECG studies as previously described^35^.

### Electrocardiographic (ECG) recordings and drug administration

For in-vivo recordings, ECG (lead II) was used in anesthetized guinea pigs (20% urethane 5 mL/kg, intraperitoneally) by placing the leads at the left forelimb as the positive electrode, the right forelimb as the negative electrode and the hindlimb as the reference electrode. The heart rate (HR), PR, QRS, QT and QTc intervals were analyzed using Bazett formula (QTc = QT/RR^1/3^, RR in s). Lyophilized azithromycin lactobionate was dissolved in saline and administrated intravenously. Hydroxychloroquine sulfate was dissolved in saline and injected intraperitoneally. Recombinant human IL-6 and tocilizumab were dissolved in saline and injected intravenously. At the completion of experiments, the deeply anaesthetized animals were sacrificed by cervical dislocation.

### Biochemical studies

#### Western blotting

Guinea pigs were injected with AZM (38.2 mg/kg) and Hydroxychloroquine (91.6 mg/kg) for 30 min and hearts were collected. Guinea pig ventricular tissue was lysed in RIPA buffer (25 mM Tris–HCl (pH 7.6), 150 mM NaCl, 1% NP-40, 1% sodium deoxycholate, 0.1% SDS)^36^. After 30 min of incubation at 4 °C, the homogenate was centrifuged at 14,000 rpm for 15 min. The supernatant after centrifugation was denatured in sample buffer for 15 min at 95 °C and then resolved by SDS-PAGE on a 4–15% Tris–HCl gel (Bio-Rad, Hercules, CA) and transferred on to a PVDF membrane (Bio-Rad, Hercules, CA). Blots were blocked with Intercept Blocking Buffer (LI-COR, Lincoln, NE) for an hour and probed with monoclonal anti-IL6Rα antibody (H-7 from Santacruz and B-R6 from Abcam) at a 1:100 dilution overnight at 4 °C. After this incubation they were washed with TBS-Tween thrice for 5 min each and further probed with secondary anti-mouse IgG secondary antibody (IRDye® 800CW) (LI-COR, Lincoln, NE) at a 1:10,000 dilution. Blots were scanned in the Odyssey CLx Imaging system (LI-COR, Lincoln, NE) at high sensitivity to obtain the image. Experiments were performed in triplicates.

### Isolation of RNA, cDNA synthesis and RT-PCR

Guinea pigs were injected with AZM (38.2 mg/kg) and HCQ (91.6 mg/kg) for 30 min and hearts were collected. Guinea pig ventricular tissue was homogenized in RLT buffer (Qiagen RNeasy fibrous tissue mini-kit) using an ultrasonic cell disruptor^37^. Total RNA was purified using RNeasy fibrous tissue mini-kit (Qiagen, Hilden, Germany). Qubit 2.0 Fluorometer (Thermo Fisher Scientific, Waltham, MA) was used to quantify RNA and to determine the purity of samples. One μg of RNA was then reverse transcribed using High-Capacity cDNA Reverse Transcription kit (Applied Biosystems, Waltham, MA). qPCR was carried out on the cDNA using TaqMan Fast Advanced Mastermix (Applied Biosystems, Waltham, MA). Genes coding for IL-6R and GAPDH were amplified on Applied Biosystem’s Quant Studio 5 PCR system. Taqman Gene Expression Assay primers were used and obtained from IDT (Integrated DNA Technologies, Coralville, IA). Primers contained the double quenched probe (5’FAM/ZEN/3’IBFQ) and ROX passive reference dye was used. Experiments were performed in triplicates.

### Electrophysiological studies

For in-vitro electrogram recordings, isolated Langendorff perfused guinea pig hearts were used. The heart from anaesthetized guinea pigs was excised, cannulated through the aorta above the coronary ostia and perfused at a constant pressure (70 mmHg) with a solution (37 °C) containing (in mM): NaCl 117, CaCl_2_ 1.8, KCl 5.7, NaHCO_3_ 4.4, MgCl_2_ 1.7, HEPES 20, NaH_2_PO_4_ 1.5, glucose 11, gassed with 95% O_2_ plus 5% CO_2_, pH7.4 adjusted with NaOH. Drugs were added in the perfusion solution via the retrograde perfusion of the coronary artery. Electrograms were obtained using a positive electrode placed at the apex, the negative electrode at the right atrium and the reference electrode at the root of the aorta. The analysis of electrogram parameters was similar to those in in-vivo experiments.

### Action potential recordings from single ventricular myocyte

Guinea pig ventricular myocytes were obtained by enzymatic dissociation. Whole-cell current clamp configuration was used to record action potential (AP) using an amplifier (Axopatch 200B, Axon Instruments) as previously described^32^. The composition of internal solution was (mM): KCl 135, EGTA 10, Glucose 5, HEPES 10, Na_2_-ATP 3, Na-GTP 0.5, pH 7.3 adjusted with KOH. The external solution contained (mM): NaCl 117, KCl 5.7, NaHCO_3_ 4.4, MgCl_2_ 1.7, HEPES 20, Glucose 20, Taurine 20, CaCl_2_ 1.8, pH 7.4 with adjusted with NaOH. All experiments were performed at room temperature (20–22 °C). APs were elicited by passing appropriate current pulses at 0.1 Hz through the recording electrode. Action potential durations at 90% full repolarization (APD_90_) and the amplitude were measured.

### Recording of L-type Ca^2+^, Na^+^ and I_Kr_ currents from guinea pig left ventricular myocytes

Whole-cell patch clamp technique was used to record L-type Ca^2+^ current (I_CaL_) current, Na^+^ current (I_Na_) and the rapid delayed rectifier current (I_Kr_) from guinea pig left ventricular myocytes^28,32^. For I_CaL_, the external solution contained (mM): NaCl 132, CsCl 5.4, CaCl_2_ 1.8, MgCl_2_ 1.8, NaH_2_PO_4_ 0.6, 4-AP 5, HEPES 10, Glucose 10, Na-pyruvate 5, pH 7.4 was adjusted with NaOH. The internal solution contained (mM): CsCl 130, MgCl_2_ 2, EGTA 11, HEPES 20, Glucose 10, Na_2_-ATP 2, Na-GTP 0.1, pH 7.3 with CsOH. To record I_CaL_, the depolarizing pulses applied from a holding potential of − 80 mV were stepped to − 40 mV for 30 ms (to inactivate I_Na_) and then to 0 mV for 300 ms at 0.1 Hz.

For I_Na_ recording, the external solution contained (mM): NaCl 70, Choline-Cl 70, KCl 5.4, MgCl_2_ 1, CaCl_2_ 0.1, HEPES 10, Glucose 10, NaH_2_PO_4_ 0.33, pH 7.4 adjusted with NaOH. The internal solution contained (mM): CsCl 120, EGTA 11, HEPES 10, Na_2_-ATP 5, MgCl_2_ 5, CaCl_2_ 1, Glucose 11, pH 7.3 with CsOH. The stimulation pulse for I_Na_ was stepped at − 40 mV for 50 ms from a holding potential of − 80 mV at 0.1 Hz.

For I_Kr_ recording, the external solution contained (mM): NaCl 145, KCl 4.5, MgCl_2_ 1, CaCl_2_ 1.8, Glucose 10, HEPES 10, pH 7.4 adjusted with NaOH. The internal solution contained (mM): KCl 140, MgCl_2_ 1, EGTA 11, HEPES 10, CaCl_2_ 1, MgATP 5, K_2_ATP 5, pH 7.30 with KOH. Currents were recorded in the whole-cell configuration of the patch-clamp technique using an Axopatch-200B amplifier (Axon Instruments, Inc, CA, USA). I_Kr_ was recorded from a holding potential (HP) of − 50 mV using a short 200 ms depolarizing pulses from − 40 to + 70 mV in a 10-mV increment before returning to − 40 mV for the tail current recording. I_CaL_ was blocked by the addition of 5 μM nifedipine in the bath solution and the slow delayed rectifier K current (I_Ks_) was blocked with 100 μM chromanol. The I_Kr_ current at + 70 mV was used for current density analysis.

### Data and statistical analyses

ECGs were analyzed using LabChart Pro software (RM6240BD, Biosignal analysis system, China) and AP and ion currents were analyzed using Sigmaplot 12.5 (Sigmaplot, Northampton, MA, USA). One-way repeated measures analysis of variance was used to compare the multiple interventions in the same preparation. Student's paired t-test was used to compare before (baseline) and after the interventions in the same preparation. Unpaired t-test was used to compare control and the interventions between independent preparations. Data are presented as means ± SE. A value of *p* < 0.05 was considered significant.

## Discussion

Here, we report a novel mechanistic explanation of the potentially fatal arrhythmogenic effect (severe bradycardia, conduction disturbances, QTc prolongation and cardiac arrest) of IL-6 in the settings of inflammation/cytokine storm in the presence of drugs known to predispose to cardiac arrhythmias. Specifically, IL-6 amplifies the arrhythmogenic impact of AZM and HCQ, and TCZ attenuated these arrhythmic events in-vivo. The observed electrocardiographic abnormalities are explained by the underlying APD prolongation and the inhibition of key ion currents (I_Na_, I_CaL_ and I_Kr_) at the single ventricular myocyte. Our data provide evidence that in the setting of inflammation characterized by high IL-6 titers, the use of medications such as AZM + HCQ increases the arrhythmogenic risk with the potential for cardiac arrest without any significant changes in IL6R gene expression by AZM + HCQ. This observation may be compounded in COVID-19 patients with the increased risk for cardiac arrhythmias secondary to acquired conditions such as the use of antiviral and other concomitant QT-prolonging drugs, fever, electrolyte imbalance, co-morbidities, and/or inherited arrhythmogenic syndromes^7–9,38^. The most important determinant of risk for malignant arrhythmias in patients with acquired QT-prolongation, is the use of one or more QTc prolonging drugs^29^ in the setting of severe manifestations of COVID-19^9^. Our data show that the combination of IL-6 + AZM + HCQ, resulted in dose-dependent bradycardia, conduction abnormalities, QTc prolongation and cardiac arrest (asystole) but without any episodes of TdP which is consistent, so far, with the clinical data from COVID-19 patients where TdP events are rare^3,39^.

To date, there is still an unmet need for understanding of the electrophysiological impact of cytokine storm-induced IL-6 overactivation in COVID-19 patients. The cytokine storm or cytokine release syndrome refers to an excessive and overwhelming release of proinflammatory mediators by an overly activated immune system^40^. Although, the immunologic mechanism of cytokine storm caused by COVID-19 is not fully understood, high IL-6 titers have been reported in COVID-19 patients^18–20,41^ and IL-6 is known to prolong APD as a result of inhibition of I_Kr_^28^, and prolong QTc interval during acute infections regardless of concomitant antimicrobial therapy^42^. APD prolongation observed with IL-6 + AZM + HCQ led to QTc prolongation thereby increasing the arrhythmic risk for COVID-19 patients. The IL-6 effect on I_Kr_ is mediated through binding to IL-6R which interacts with membrane-bound gp130 and its downstream Janus-kinase signaling pathways^28,43^. In support of the role of proinflammatory IL-6 in COVID-19 patients, TCZ which is a recombinant humanized monoclonal anti-IL-6R antibody approved by FDA for the treatment of severe or life-threatening chimeric antigen-receptor-T-cell-induced cytokine release syndrome^44^, is being tested in COVID-19 patients^21–24^. Our data demonstrating that TCZ prevented the electrocardiographic abnormalities induced by IL-6 + AZM + HCQ suggest that targeting key molecules within the inflammatory cytokine storm such as IL-6, could be a novel strategy for limiting the direct electrophysiological and arrhythmogenic effects of IL-6 in COVID-19 patients^26^ and beyond. Accordingly, two clinical studies demonstrated the effectiveness of TCZ in rapidly reversing QTc prolongation in rheumatoid arthritis patients, by controlling systemic inflammation^45,46^.

HCQ has an established safety profile at appropriate doses and is used to treat malaria, and autoimmune diseases such as rheumatoid arthritis and lupus erythematosus^47,48^. Because HCQ demonstrated antiviral activity^49^ and ability to regulate the immune system^50^, it was assumed that it may be useful in the treatment of COVID-19. Based on initial favorable outcomes from a small number of non-randomized clinical studies^17,51,52^, FDA had issued an emergency use authorization to HCQ for hospitalized COVID-19 patients on March 28, 2020, to just revoke this authorization on June 15th 2020 based a randomized double blind HCQ clinical study which showed no additional benefit for COVID-19 patients compared to placebo control^12,53^. The macrolid antibiotic drug AZM is employed in the clinical practice worldwide to treat different types of common bacterial infections, more frequently pneumonia and other respiratory infections. More recently, AZM has been used in combination with HCQ for the treatment of COVID-19 but there was no consensus on the clinical outcomes with some studies demonstrating that the combination of AZM + HCQ resulted in a favorable clinical outcomes^17,52^ and others reporting arrhythmia safety concerns^4,7,15^. The risk of QTc prolongation and TdP has been reported for both HCQ and AZM when used alone^54^ but QTc prolongation seems to be the major concern when these drugs are used in combination, specifically in COVID-19 patients^39,55^. In fact, several recent studies reported that this combination therapy was associated with significant QTc lengthening (on average ~ 25-35 ms increase when compared to baseline) and high rates of marked QTc prolongation > 500 ms (~ 10–35% of treated subjects^39,56,57^). Conversely, in a previous study involving 116 healthy controls, co-administration of AZM(500–1,500 mg/day) + chloroquine(1,000 mg/day) induced only mild QTc lengthening(5-9 ms)^58^, thereby supporting a key role for inflammatory activation, specifically IL-6 in boosting QT-prolonging potential of these drugs. In agreement with these findings are our in-vivo experiments demonstrating that while the combination of IL-6 + AZM + HCQ resulted in a marked QTc prolongation, AZM + HCQ when administered alone without IL-6, only mild and not significant QTc changes were observed.

While only rare cases of TdP have been reported in COVID-19 patients^3,56,59^, other arrhythmic episodes included cardiac arrest, significant bradyarrhythmias, and non-sustained ventricular tachycardia were also reported^3^. These clinical findings are consistent with our observation that the combination of IL-6 + AZM + HCQ, but not AZM + HCQ, results in marked bradycardia, QTc prolongation and cardiac arrest (asystole). Bradycardia and conduction disturbances are likely due to HCQ inhibition of the “funny” current, I_f_^31^ and/or AZM inhibition of I_CaL_^32^. With the concentration of 200 µg/L of IL-6, we observed an inhibition of I_CaL_ which may compound the effects of AZM in causing further bradycardia and atrioventricular conduction abnormalities. The observed QTc prolongation with the combination of IL-6 + AZ + HCQ both in-vivo and in-vitro is due to the inhibition of I_Kr_, which resulted in the prolongation of ventricular-myocyte AP and QT interval. Because the combination of IL-6 + AZM + HCQ also inhibited I_CaL_, this is expected to counterbalance the extent of AP prolongation and hence QTc. This multi-channel affinity with opposing effects may, in part, explain the absence or rarity of TdP arrhythmia. The combination of IL-6 + AZM + HCQ also inhibited I_Na_ likely resulting, together with the concomitant I_CaL_ inhibition, in conduction abnormalities like PR interval prolongation. It is also plausible that I_Na_ inhibition in addition to I_CaL_ inhibition may have both reduced susceptibility to TdP. In this regard, it has been previously proposed that multi-channel effects must be considered when evaluating the TdP risk^29^. Notably, beyond the combination therapy for COVID-19, AZM and HCQ are frequently employed also uncombined, still always during active inflammatory processes, i.e. acute bacterial infections or immune-inflammatory diseases, respectively. Based on the above mechanistic considerations, it is very likely that also in these pathologic conditions IL-6 could increase the arrhythmogenic potential of these drugs, even critically if, as commonly occurs, other pro-arrhythmic medications and/or non-pharmacologic risk factors are concomitantly present.

While guinea pigs exhibit the advantage of having an ECG and AP morphology similar to humans, the findings from this study may not be similar in human especially in the presence of a particular pathology like COVID-19 where often other comorbidities are concomitant. Consistent with our data is the recent work from Li et al.^60^, demonstrating that the clinically observed QT prolongation caused by treatment with HCQ could be recapitulated in human induced pluripotent stem cells derived cardiomyocytes (iPSC-CMs) by measuring field potential duration (FPDc) which is a surrogate for an electrocardiogram from cell monolayer. The authors showed that HCQ-induced FPDc prolongation was markedly enhanced by the combined treatment with AZM in iPSC-CMs. The authors have not examined the effects of IL-6 on HCQ + AZM. The fact that we could not induce any episodes of TdP in these healthy guinea pigs, does not mean that TdP cannot be triggered by the combination of an inflammatory state (IL-6) and the use of both AZM and HCQ in humans. Nevertheless, our animal data is consistent with several clinical reports demonstrating that TdP is not frequent in COVID-19 patients and that more in general in the setting of inflammation, IL-6 is a potential additional non-conventional risk factor for the development of cardiac arrhythmias alone or in combination with antimalarial or antibiotic drugs.

## Conclusions

In the settings of severe infection, high levels of IL-6 combined with AZM and HCQ, can cause fatal arrhythmias and cardiac arrest via excessive conduction abnormalities, prolongation of the QTc and APD as a result of the multi-channels’ inhibition of I_Na_, I_CaL_ and I_Kr_. HCQ continues to be used in several countries for COVID-19 patients^14^, and for malaria and for autoimmune diseases such as lupus erythematosus and rheumatoid arthritis both conditions known to also experience high levels of IL-6 along with increased prevalence of QTc prolongation and sudden cardiac death^61–63^. In addition, there seems to be a renewed interest in the use of HCQ not only for COVID-19 patients but also as a prophylaxis for asymptomatic COVID-19 subjects as well as healthcare professionals^14,64^. Our findings are clinically relevant in assessing the arrhythmic risk when HCQ is combined with the commonly used AZM or other QTc prolonging factors during infection and/or inflammation. Intriguingly, the enhancer role demonstrated here for IL-6 on the arrhythmogenic potential associated with HCQ/AZM treatment, could be extended to many other medications, particularly those listed at crediblemeds.org as QT-prolonging drugs. In fact, similarly to HCQ and AZM, it is well-recognized that most of these molecules can delay ventricular repolarization by blocking I_Kr_, in turn representing the key electrophysiological mechanism underlying IL-6-mediated QTc prolongation. In this way, COVID-19 could provide the opportunity to more generally decipher drug-induced arrhythmogenesis during inflammatory processes.

Finally, as we move forward with COVID-19 pandemic, and in order to reduce mortality and for better preparedness, there continue to be an urgent need for the understanding of the electrophysiologic mechanism of drug combinations and the discovery of novel drug combination with a safe arrhythmic profile.

## Supplementary Information

Supplementary Legends.

Supplementary Figure S1.

## References

[1] Medicine, J. H. U. Coronavirus Resource Center. https://coronavirus.jhu.edu/ (2021).

[2] Organization, W. H. https://www.who.int/emergencies/diseases/novel-coronavirus-2019/situation-reports. (2021).

[3] Bhatla A (2020). COVID-19 and cardiac arrhythmias. Heart Rhythm.

[4] Lakkireddy DR (2020). Guidance for rebooting electrophysiology through the COVID-19 pandemic from the Heart Rhythm Society and the American Heart Association Electrocardiography and Arrhythmias Committee of the Council on Clinical Cardiology: Endorsed by the American College of Cardiology. Heart Rhythm.

[5] Beri A, Kotak K (2020). Cardiac injury, arrhythmia, and sudden death in a COVID-19 patient. HeartRhythm Case Rep..

[6] Kochav SM (2020). Cardiac Arrhythmias in COVID-19 Infection. Circ. Arrhythm. Electrophysiol..

[7] Giudicessi JR, Noseworthy PA, Friedman PA, Ackerman MJ (2020). Urgent guidance for navigating and circumventing the QTc-prolonging and torsadogenic potential of possible pharmacotherapies for coronavirus disease 19 (COVID-19). Mayo Clin. Proc..

[8] Roden DM, Harrington RA, Poppas A, Russo AM (2020). Considerations for drug interactions on QTc in exploratory COVID-19 treatment. Circulation.

[9] Lazzerini PE, Boutjdir M, Capecchi PL (2020). COVID-19, Arrhythmic Risk, and Inflammation: Mind the Gap!. Circulation.

[10] Malviya A (2020). Ventricular arrhythmia risk due to chloroquine/hydroxychloroquine treatment for COVID-19: Should it be given. Indian Heart J..

[11] Pereira BB (2020). Challenges and cares to promote rational use of chloroquine and hydroxychloroquine in the management of coronavirus disease 2019 (COVID-19) pandemic: A timely review. J. Toxicol. Environ. Health B Crit. Rev..

[12] Kapoor A (2020). Cardiovascular risks of hydroxychloroquine in treatment and prophylaxis of COVID-19 patients: A scientific statement from the Indian Heart Rhythm Society. Indian Pac. Electrophysiol. J..

[13] Uzelac I (2020). Fatal arrhythmias: Another reason why doctors remain cautious about chloroquine/hydroxychloroquine for treating COVID-19. Heart Rhythm.

[14] Global HCQ Studies. https://c19study.com (2021).

[15] Sarayani A, Cicali B, Henriksen CH, Brown JD (2021). Safety signals for QT prolongation or Torsades de Pointes associated with azithromycin with or without chloroquine or hydroxychloroquine. Res. Soc. Adm. Pharm..

[16] Sivapalan P (2020). Proactive prophylaxis with azithromycin and hydroxychloroquine in hospitalised patients with COVID-19 (ProPAC-COVID): A structured summary of a study protocol for a randomised controlled trial. Trials.

[17] Gautret P (2020). Clinical and microbiological effect of a combination of hydroxychloroquine and azithromycin in 80 COVID-19 patients with at least a six-day follow up: A pilot observational study. Travel Med. Infect. Dis..

[18] Zhou F (2020). Clinical course and risk factors for mortality of adult inpatients with COVID-19 in Wuhan, China: A retrospective cohort study. Lancet.

[19] Huang C (2020). Clinical features of patients infected with 2019 novel coronavirus in Wuhan, China. Lancet.

[20] Chen N (2020). Epidemiological and clinical characteristics of 99 cases of 2019 novel coronavirus pneumonia in Wuhan, China: A descriptive study. Lancet.

[21] Luo P (2020). Tocilizumab treatment in COVID-19: A single center experience. J. Med. Virol..

[22] Xu X (2020). Effective treatment of severe COVID-19 patients with tocilizumab. Proc. Natl. Acad. Sci. U S A.

[23] Farooqi F (2020). Treatment of severe COVID-19 with tocilizumab mitigates cytokine storm and averts mechanical ventilation during acute respiratory distress: A case report and literature review. Trop. Med. Infect. Dis..

[24] ElSeirafi MM (2020). Efficacy and safety of tocilizumab in critically ill adults with COVID-19 infection in Bahrain: A report of 5 cases. Respir. Med. Case Rep..

[25] Atal S, Fatima Z (2020). IL-6 inhibitors in the treatment of serious COVID-19: A promising therapy?. Pharmaceut. Med..

[26] Lazzerini PE, Laghi-Pasini F, Acampa M, Boutjdir M, Leopoldo Capecchi P (2020). IL-6 (Interleukin 6) blockade and heart rate corrected QT interval prolongation in COVID-19. Circ. Arrhythm. Electrophysiol..

[27] Lazzerini PE (2017). Systemic inflammation as a novel QT-prolonging risk factor in patients with torsades de pointes. Heart.

[28] Aromolaran AS (2018). Interleukin-6 inhibition of hERG underlies risk for acquired long QT in cardiac and systemic inflammation. PLoS ONE.

[29] Lazzerini PE, Capecchi PL, El-Sherif N, Laghi-Pasini F, Boutjdir M (2018). Emerging arrhythmic risk of autoimmune and inflammatory cardiac channelopathies. J. Am. Heart Assoc..

[30] Lazzerini PE, Laghi-Pasini F, Boutjdir M, Capecchi PL (2019). Cardioimmunology of arrhythmias: The role of autoimmune and inflammatory cardiac channelopathies. Nat. Rev. Immunol..

[31] Capel RA (2015). Hydroxychloroquine reduces heart rate by modulating the hyperpolarization-activated current If: Novel electrophysiological insights and therapeutic potential. Heart Rhythm.

[32] Zhang M (2017). Electrophysiologic studies on the risks and potential mechanism underlying the proarrhythmic nature of azithromycin. Cardiovasc. Toxicol..

[33] Reagan-Shaw S, Nihal M, Ahmad N (2008). Dose translation from animal to human studies revisited. FASEB J..

[34] Chen L (2012). Confirmation of a proarrhythmic risk underlying the clinical use of common Chinese herbal intravenous injections. J. Ethnopharmacol..

[35] Nellan A (2018). Improved CNS exposure to tocilizumab after cerebrospinal fluid compared to intravenous administration in rhesus macaques. Blood.

[36] Yue Y (2015). Pathogenesis of the novel autoimmune-associated long-QT syndrome. Circulation.

[37] Srivastava U (2020). Novel re-expression of L-type calcium channel Cav1.3 in left ventricles of failing human heart. Heart Rhythm.

[38] Wu CI (2020). SARS-CoV-2, COVID-19, and inherited arrhythmia syndromes. Heart Rhythm.

[39] Chorin E (2020). The QT interval in patients with COVID-19 treated with hydroxychloroquine and azithromycin. Nat. Med..

[40] Lee DW (2014). Current concepts in the diagnosis and management of cytokine release syndrome. Blood.

[41] Fang Y (2020). CT Manifestations of two cases of 2019 novel coronavirus (2019-nCoV) pneumonia. Radiology.

[42] Lazzerini PE (2020). Cardiac arrest risk during acute infections: Systemic inflammation directly prolongs QTc interval via cytokine-mediated effects on potassium channel expression. Circ. Arrhythm Electrophysiol..

[43] Johnson DE, O'Keefe RA, Grandis JR (2018). Targeting the IL-6/JAK/STAT3 signalling axis in cancer. Nat. Rev. Clin. Oncol..

[44] Le RQ (2018). FDA approval summary: Tocilizumab for treatment of chimeric antigen receptor t cell-induced severe or life-threatening cytokine release syndrome. Oncologist.

[45] Lazzerini PE (2015). Antiarrhythmic potential of anticytokine therapy in rheumatoid arthritis: Tocilizumab reduces corrected QT interval by controlling systemic inflammation. Arthritis Care Res. (Hoboken).

[46] Kobayashi H (2018). Heart rate-corrected QT interval duration in rheumatoid arthritis and its reduction with treatment with the interleukin 6 inhibitor tocilizumab. J. Rheumatol..

[47] Ben-Zvi I, Kivity S, Langevitz P, Shoenfeld Y (2012). Hydroxychloroquine: From malaria to autoimmunity. Clin. Rev. Allergy Immunol..

[48] Plantone D, Koudriavtseva T (2018). Current and future use of chloroquine and hydroxychloroquine in infectious, immune, neoplastic, and neurological diseases: A mini-review. Clin. Drug Investig..

[49] Yao X (2020). In vitro antiviral activity and projection of optimized dosing design of hydroxychloroquine for the treatment of severe acute respiratory syndrome coronavirus 2 (SARS-CoV-2). Clin. Infect. Dis..

[50] Martinez GP, Zabaleta ME, Di Giulio C, Charris JE, Mijares MR (2020). The role of chloroquine and hydroxychloroquine in immune regulation and diseases. Curr. Pharm. Des..

[51] Gao J, Tian Z, Yang X (2020). Breakthrough: Chloroquine phosphate has shown apparent efficacy in treatment of COVID-19 associated pneumonia in clinical studies. Biosci. Trends.

[52] Gautret P (2020). Hydroxychloroquine and azithromycin as a treatment of COVID-19: Results of an open-label non-randomized clinical trial. Int. J. Antimicrob. Agents.

[53] Self WH (2020). Effect of hydroxychloroquine on clinical status at 14 days in hospitalized patients with COVID-19: A randomized clinical trial. JAMA.

[54] Teng C, Walter EA, Gaspar DKS, Obodozie-Ofoegbu OO, Frei CR (2019). Torsades de pointes and QT prolongation associations with antibiotics: A pharmacovigilance study of the FDA adverse event reporting system. Int. J. Med. Sci..

[55] Hsia BC (2020). QT prolongation in a diverse, urban population of COVID-19 patients treated with hydroxychloroquine, chloroquine, or azithromycin. J. Interv. Card Electrophysiol..

[56] Chorin E (2020). QT interval prolongation and torsade de pointes in patients with COVID-19 treated with hydroxychloroquine/azithromycin. Heart Rhythm.

[57] Bessiere F (2020). Assessment of QT intervals in a case series of patients with coronavirus disease 2019 (COVID-19) infection treated with hydroxychloroquine alone or in combination with azithromycin in an intensive care Unit. JAMA Cardiol..

[58] Hancox JC, Hasnain M, Vieweg WV, Crouse EL, Baranchuk A (2013). Azithromycin, cardiovascular risks, QTc interval prolongation, torsade de pointes, and regulatory issues: A narrative review based on the study of case reports. Ther. Adv. Infect. Dis..

[59] Anupama BK, Adhikari S, Chaudhuri D (2020). Prolonged QT interval in a patient with coronavirus disease-2019: Beyond hydroxychloroquine and azithromycin. J. Investig. Med. High Impact Case Rep..

[60] Li W, Luo X, Poetsch M, Oertel R, Nichani K, Schneider M, Strano A, Hasse M, Steiner RP, Cyganek L, Hettwer K, Uhlig S, Simon K, Guan K, Schubert M (2021). Effects of hydroxychloroquine and azithromycin on iPSC-derived cardiomyocytes: Considerations for the treatment of COVID-19 patients. bioRxiv.

[61] Lazzerini PE, Capecchi PL, Laghi-Pasini F (2015). Long QT syndrome: An emerging role for inflammation and immunity. Front Cardiovasc. Med..

[62] Davis LS, Hutcheson J, Mohan C (2011). The role of cytokines in the pathogenesis and treatment of systemic lupus erythematosus. J. Interferon. Cytokine Res..

[63] Lazzerini PE (2016). Marked QTc prolongation and torsades de pointes in patients with chronic inflammatory arthritis. Front Cardiovasc. Med..

[64] Pulla P (2020). India expands use of controversial drug for coronavirus despite safety concerns. Nature.

