# Supplementary figures and images for: Arrhythmogenic mechanisms of interleukin-6 combination with hydroxychloroquine and azithromycin in inflammatory diseases
Source: Sci Rep. 2022 Jan 20;12:1075. doi: 10.1038/s41598-022-04852-5 (PMC8776801; doi:10.1038/s41598-022-04852-5)

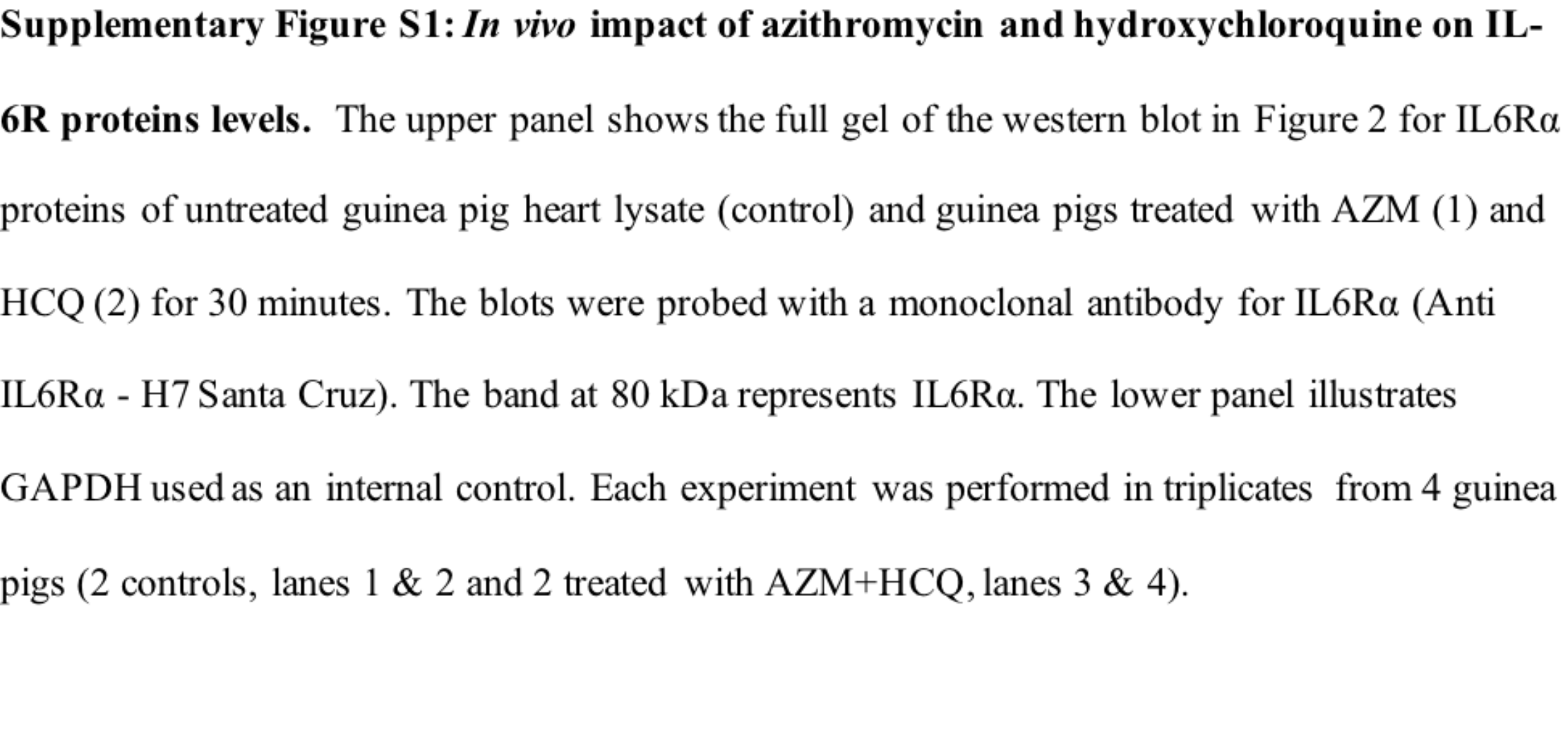

Supplement: Supplementary file 1 — Supplementary Legends. [file 41598_2022_4852_MOESM1_ESM.tif]

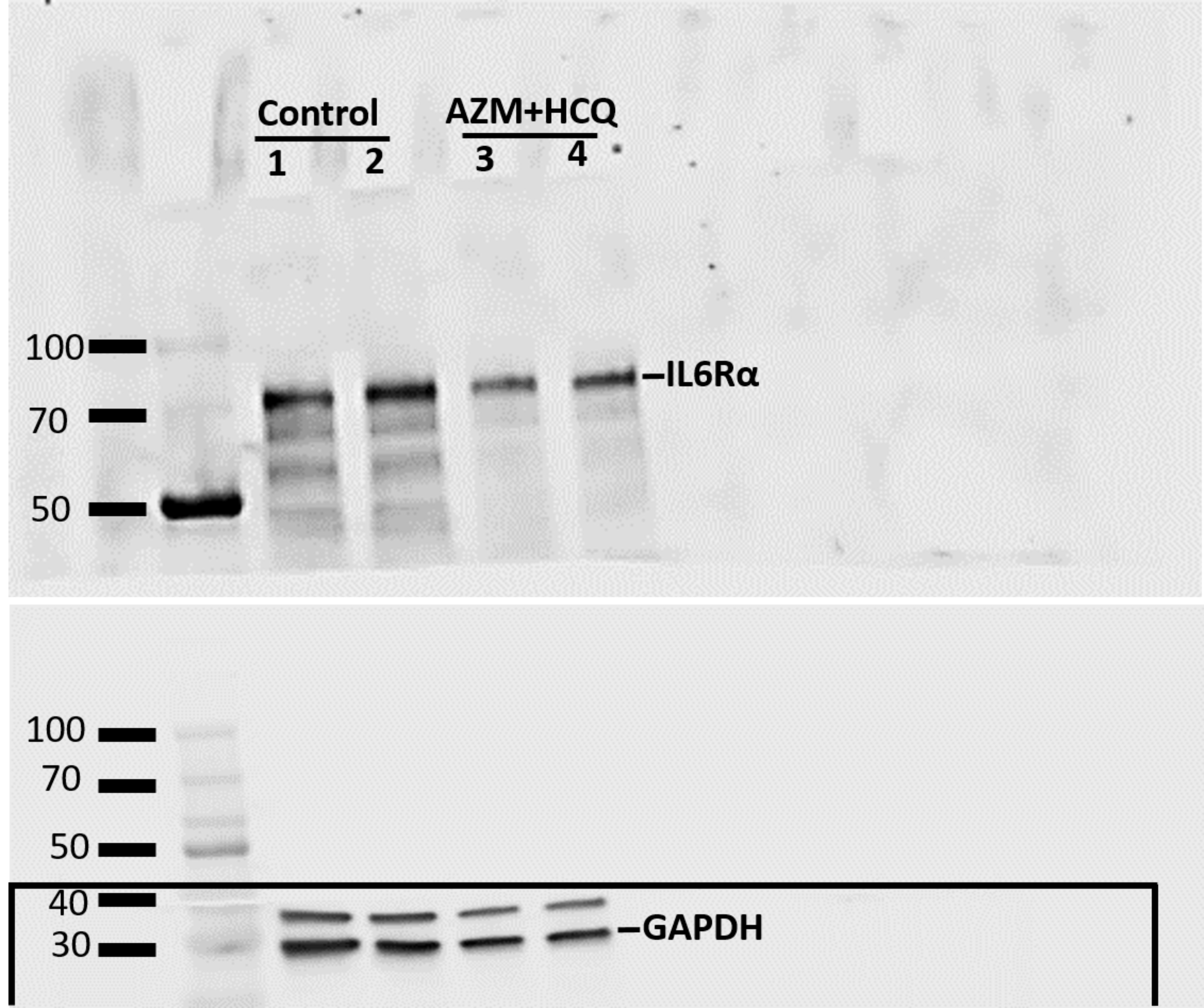

Supplement: Supplementary file 2 — Supplementary Figure S1. [file 41598_2022_4852_MOESM2_ESM.tif]
